# Medical-Economic and Ecological Impact of Anesthesia Teleconsultation: Retrospective Observational Study

**DOI:** 10.2196/70259

**Published:** 2025-11-13

**Authors:** Fabrice Ferre, Philippine Furelau, François Labaste, Nadège Costa, Fanny Vardon, Antoine Piau, Charlotte Martin, Vincent Minville

**Affiliations:** 1 Centre Hospitalier Universitaire de Toulouse Toulouse France

**Keywords:** teleconsultation, telemedicine, anesthesia, economic, ecological, carbon impact, carbon dioxide equivalent, CO2eq, greenhouse gas

## Abstract

**Background:**

Telemedicine, particularly teleconsultation, has emerged as a viable alternative to in-person consultation, especially following the COVID-19 pandemic. Preanesthetic consultations are mandatory before surgery to assess perioperative risk. However, little data exists regarding the combined economic and ecological impacts of replacing in-person consultation with teleconsultation in this context.

**Objective:**

The primary aim was to evaluate the financial and environmental benefits of teleconsultation for preanesthetic consultation. Secondary objectives included assessing patient satisfaction and perioperative safety.

**Methods:**

This retrospective, single-center observational study included patients scheduled for orthopedic surgery between September 2020 and October 2020 at Toulouse University Hospital. Eligible patients completed a preconsultation questionnaire via the MyAnesth digital agent. Patients were allocated to teleconsultation or in-person consultation groups based on predefined criteria. Postoperative data on demographics, transportation, consultation modality, time off work, and patient satisfaction were collected. Economic analysis included travel costs, income loss, and health insurance reimbursements. Ecological analysis quantified greenhouse gas (GHG) emissions based on transportation mode and digital infrastructure use. Statistical comparisons between the teleconsultation and in-person consultation groups used appropriate parametric and nonparametric tests, with significance set at *P*≤.05.

**Results:**

A total of 401 patients were analyzed (teleconsultations: n=331, 82.5%; in-person consultations: n=70, 17.5%). Teleconsultations reduced the average travel distance by 46,000 km, corresponding to 9.7 tons of carbon dioxide equivalent saved. Mean cost savings per patient were €122 (SD €125; 1 US $=€1.17), with total savings of €42,840 for patients and the national health care system. Teleconsultations also significantly reduced time spent on travel and administrative processes (mean 22, SD 9 minutes vs mean 130, SD 16 minutes for in-person consultations; *P*<.001). No significant differences in postoperative complication rates were observed between groups (teleconsultations: 11/331, 3.3%; in-person consultations: 5/70, 7.1%; *P*=.24). Patient satisfaction scores were high and similar in both groups (median 9, IQR 8-10, of a possible 10), with most patients preferring teleconsultations or expressing no preference for consultation modality. Digital teleconsultation infrastructure contributed minimally to GHG emissions (2.3 kg of carbon dioxide equivalent for 331 teleconsultations), representing a 99% reduction compared to travel-based in-person consultations.

**Conclusions:**

Teleconsultations for preanesthetic assessment demonstrated significant economic and ecological advantages without compromising clinical safety or patient satisfaction. Patients reported high levels of satisfaction and minimal attachment to in-person consultations and appreciated the convenience of remote access. This model reduces unnecessary travel, limits health care–related GHG emissions, and generates considerable cost savings for both patients and public health systems. These findings support broader integration of teleconsultations into routine anesthetic care, particularly for low-risk outpatient surgical candidates. Expanding teleconsultation eligibility criteria could enhance health care system efficiency and contribute to sustainable medical practice.

## Introduction

### Background

Telemedicine is the remote diagnosis, treatment, and monitoring of patients using telecommunications technology. In 1997, the World Health Organization officially recognized telemedicine for the first time, defining it as “activities, services, and health systems conducted remotely using information and communication technologies to meet global health promotion, care, epidemic control, management, and health-related research needs [1].” This global endorsement of telemedicine and the development of suitable communication tools marked the beginning of a new era of “modern” telemedicine. In this context, the development of teleconsultation facilitated health care access in underserved areas, reduced health care avoidance [2], shortened wait times [3], and improved patient follow-up [4]. A sudden acceleration in the use of telemedicine occurred in March 2020, driven by the COVID-19 pandemic [5,6]. Since then, teleconsultation has experienced a significant rise, becoming widespread across numerous specialties and leading to an exponential increase in its use, even in pediatric and obstetric populations [7,8]. The preanesthetic consultation (PAC) is a mandatory step before any scheduled surgery. Its purpose is to assess the patient’s health status, determine the need for additional examinations or specialized consultations, and evaluate the risk of complications during the perioperative period. Beyond reducing interpersonal contact, as required during a pandemic, telemedicine decreases the patient travel costs and associated expenses generated by in-person consultations for both patients and health care systems [6]. As such, teleconsultations could offer significant medical-economic advantages [9,10]. In parallel with digital progress, the health care sector is beginning to participate in the ongoing ecological revolution. The “forced” replacement of in-person consultations with teleconsultations during the COVID-19 pandemic highlighted the feasibility of maintaining care quality while reducing unnecessary travel [6,11]. To our knowledge, no data deal with the real economic and ecological effect of teleconsultations. Several studies have evaluated the benefits of teleconsultation in various medical specialties, demonstrating improved accessibility, patient satisfaction, and potential cost savings. However, these studies have rarely quantified the combined economic and environmental impacts of replacing in-person consultations with teleconsultations. This study aimed to fill this gap by evaluating both the financial and environmental consequences of implementing teleconsultations for PACs.

### Objectives

Thus, the primary objective of this study was to evaluate the economic and ecological benefits generated by conducting preanesthesia teleconsultations. Secondary objectives included assessing patient satisfaction and safety in preanesthetic teleconsultations.

## Methods

### Experimental Design

This was a retrospective, observational, single-center study conducted in the orthopedic surgery anesthesia department at Toulouse University Hospital.

### Ethical Considerations

This research is considered an experiment in educational sciences aiming to evaluate the participative and pedagogical quality of a new digital tool implemented in current practice.

This study was exempt from formal institutional review board approval according to French regulations as it involved retrospective analysis of anonymized data collected during routine care, with no intervention or identifiable personal data. As such, it did not fall within the scope of research involving human participants as defined by the French Public Health Code (Articles L1121-1 and R1121-1). Therefore, no ethics committee review was required. This study did not present any risk for the participants and did not modify the usual care pathway or the time required for patient management.

### Study Population

All patients scheduled for a PAC for planned orthopedic surgery between September 2020 and October 2020 who responded to the digital conversational agent MyAnesth to prepare for their PAC were eligible. The digital conversational agent MyAnesth was developed in collaboration with a company that creates secure health companions (BOTdesign) [12]. Medical data obtained using MyAnesth allowed for the calculation of a numerical score (MyRISK score) for each patient, stratifying them into low (green), intermediate (orange), or high (red) perioperative risk [13]. Exclusion criteria included the conduct of a relocated PAC (ie, conducted at another hospital center), the presence of a legal guardianship or protection regime (guardianship, trusteeship, or judicial protection), patients who did not speak French, and patients who opposed participation in the study.

### PAC Modalities

The modality for the anesthesia consultation (in-person consultation or teleconsultation) was decided based on predefined criteria. Eligibility criteria for teleconsultations included patient consent, a surgical intervention considered minor or intermediate (eg, knee arthroscopy, hand or foot surgery, or removal of osteosynthesis material), good comprehension of French, and sufficient cognitive and technical capabilities for teleconsultation (adequate equipment). Thus, 2 preanesthetic trajectories were identified, defining the teleconsultation and in-person consultation groups. As per our standard of care, the allocated time for the PAC was 20 minutes.

Teleconsultations were conducted via the TéléO platform (NEHS DIGITAL) and created by the National Mutual of Hospital Workers. TéléO has been used at Toulouse University Hospital since the onset of the COVID-19 pandemic (March 2020). The platform features secure connectivity with internet speed tests and audio and video quality verification. The application is described as “full web,” operating solely with an internet connection through any browser. TéléO operates on Web Real-Time Communication, a technology that allows applications and websites to exchange voice calls and video chats and conduct instant file sharing without requiring the user to install plug-ins or other third-party software. TéléO is accessible from any device (smartphone, tablet, desktop, or laptop computer).

### Postoperative Data Collection

Patients were recontacted via phone postoperatively by one of the anesthesia team investigators to collect the following study-related data:

- Demographic characteristics, including age, sex, type of surgery, and American Society of Anesthesiologists score.
- PAC modality (in-person consultation or teleconsultation), including mode of transportation used to attend the in-person consultation (car, medical transport, public transportation, bicycle, or scooter), distance between the home and the hospital, presence of a companion, and whether the patient or companion needed to take a full or half day off from work. For this purpose, patients who had undergone teleconsultations were asked to answer these questions hypothetically as if they had attended an in-person consultation (“If you had attended in person...”).
- Patient satisfaction with the PAC, collected using a simple numerical scale ranging from 0 (“extremely dissatisfied”) to 10 (“extremely satisfied”). Patients were also asked about their preference for a future PAC modality (teleconsultation, in-person consultation, or no preference).

All data were anonymized.

### Economic Data Collection

#### Concerning Patients and Health Insurance

##### Transportation Costs

Transportation costs by car were estimated based on the standard mileage allowance for vehicle use (order of February 15, 2021; [14]) using the formula distance × 0.541 (average for a vehicle with horsepower of 60 to 150). This rate includes vehicle power, fuel consumption, depreciation, maintenance costs, tire wear, and insurance premiums.

Transportation costs for medical vehicles were calculated based on the applicable rates for the Occitania region [15]. These include a base fee (averaged at €12.70; a conversion rate of 1 US $=€1.17 applies), a patient pickup fee (€15.58), and a per-kilometer rate (€1.02). The patient’s copay was €4.00 for a round trip. Short-distance fees (under 18 km) and additional fees for ambulance transport were not considered to avoid overestimating results. Health insurance was assumed to reimburse 65%.

##### Salary Costs

Time off work for patients or their companions to attend the PAC was recorded and considered as vacation or compensatory time off. Sick leave and employer or health insurance coverage were not considered due to the 1-day waiting period since 2018.

The loss of income for patients was calculated based on the following:

- The French median gross hourly wage in 2018, €15.20 per hour (National Institute of Statistics and Economic Studies 2021), including base salary and overtime or bonuses.
- The average French working hours (National Institute of Statistics and Economic Studies 2019 survey). The average workday is 7.9 hours for full-time employees and 8.4 hours for nonemployees, with a national distribution of 87.9% employees and 12.1% self-employed.

For retirees or unemployed patients, the loss of leisure time was evaluated at 33% of the net salary.

##### Connection Costs

For teleconsultations, internet or device costs were not included if most patients already had internet access for personal use in 2020.

##### Travel and Consultation Time

Travel times and distances between patients’ homes and the hospital were calculated using Google Maps based on the postal code provided during administrative registration. Cycling speed was estimated at 15 km/h. Public transportation times were not included due to excessive variability. The average time spent at the hospital from administrative check-in was calculated from a sample of 30 in-person consultation patients. For the teleconsultation group, transportation costs and times were extrapolated from patient responses regarding hypothetical in-person consultation attendance.

#### Concerning the Hospital

##### Software Investments

The TéléO application was funded by the Regional Health Agency and made available free of charge throughout the region.

##### Anesthesia Consultation

Under activity-based pricing, the price of a teleconsultation was set at €23 (specialized consultation, hospital billing guide 2020). The average duration of the PAC was calculated from a sample of 60 patients (30 for teleconsultations and 30 for in-person consultations).

### Ecological Data Collection

Greenhouse gas (GHG) emissions were estimated based on (1) activity data (energy consumption in kilowatt-hours and distances traveled in kilometers) and (2) carbon emission factors (from the Environment and Energy Management Agency (Agence De l’Environnement et de la Maîtrise de l’Énergie) carbon database, representing the quantity of GHGs emitted by the activity). Results were expressed in tons of carbon dioxide equivalent (CO_2_eq).

#### Transportation Emissions

For passenger vehicles, a medium-sized car with various types of engines (excluding electric and hybrid) was considered, with an emission factor of 0.218 kg CO_2_eq per kilometer. Medical transport was considered to have the same emission factor. Public transport emissions were calculated assuming metro or tramway use, with an average factor of 0.0024 kg CO_2_eq per kilometer. Bus use was not considered to avoid overestimating results. Our decision to exclude the bus was based on the following rationale: overlap with other modes. In the study area, bus routes often run parallel to tram and metro lines. This study primarily focused on high-capacity, fixed-route modes (metro and tram) due to their greater relevance for structural transport planning. Buses, being more flexible and variable, would have required a different modeling approach. Flights were considered short haul, with an emission factor of 0.414 kg CO_2_eq per kilometer.

The carbon dioxide emission factors used in this study were taken from the carbon database of the Agence De l’Environnement et de la Maîtrise de l’Énergie [16], which provides official French emission data for various transport modes and energy sources. Cost assumptions were based on national sources, including the Centre for Studies and Expertise on Risks, the Environment, Mobility, and Urban Planning (2020) for unit transport costs [17].

#### Teleconsultation Emissions

Energy consumption from IT equipment using fiber and digital subscriber lines was considered. A 5-minute connection to TéléO and a 20-minute teleconsultation session were assumed. Equipment used for teleconsultations included a fully equipped desktop computer (monitor, speakers, webcam, printer, and internet) consuming 200 W (Wh) per hour of use, equivalent to 67 Wh for 20 minutes. Patient equipment energy consumption was only considered when using a computer (assumed to be a desktop) to avoid overestimation. Energy consumption from smartphones or tablets was not included as their use primarily affects manufacturing emissions rather than use.

### Statistical Analysis

Qualitative data were expressed as percentages. Normality of data was tested using the Shapiro-Wilk test. Quantitative data were expressed as medians and IQRs or means and SDs. Qualitative variables were compared using the Fisher exact test or chi-square test, and quantitative variables were compared using the Mann-Whitney *U* test. Statistical analysis was conducted using the MedCalc software (version 12.6.1; MedCalc Software Ltd) and the online statistics platform pvalue.io (Medistica). A *P* value of ≤.05 was considered statistically significant.

## Results

### Patient Characteristics

Of the 423 patients included, 401 (94.8%) completed the survey, enabling the analysis of study-related end points. The distribution of patients between the teleconsultation and in-person consultation groups is presented in Table 1. The mean age was 41.4 (SD 16.2) years. Most surgical interventions were considered minor (292/401, 72.8%) and scheduled as outpatient procedures (304/401, 75.8%). Patients seen via teleconsultation were younger, professionally active, and scheduled for minor outpatient surgeries. The primary connection device used was a computer (245.331, 73.6%), followed by a smartphone (53/331, 16%) and a tablet (27/331, 8.2%). A total of 17.5% (70/401) of all patients had previously experienced teleconsultations.

**Table 1:** Patient characteristics (N=401).a

		Total	Teleconsultations (n=331)	In-person consultations (n=70)	*P* value	
**Sex, n (%)**						.35
	Male	238 (59.4)	193 (58.3)	45 (64.3)		
	Female	163 (40.6)	138 (41.7)	25 (35.7)		
**Age (y), n (%)**						
	≤25	67 (16.7)	70 (21.1)	7 (10)		
	26-45	162 (40.4)	132 (39.9)	29 (41.4)		
	46-65	129 (32.2)	109 (32.9)	21 (30)		
	>65	43 (10.7)	20 (6)	13 (18.6)		
Age (y), mean (SD)		41.4 (16.2)	40.4 (15.6)	48.0 (17.3)	*<.001*	
Age (y), median (IQR)		40 (28-55)	38 (27.0-54.0)	46.5 (33.5-62.0)		
**ASA** ^b^ **score, n (%)**						*<.001*
	1	289 (72.1)	250 (75.5)	36 (51.4)		
	2	93 (23.2)	69 (20.8)	24 (34.3)		
	3	19 (4.7)	9 (2.7)	10 (14.3)		
	4	0 (0)	0 (0)	0 (0)		
**MyRISK score, n (%)**						.23
	Green	106 (26.4)	93 (28.1)	13 (18.6)		
	Orange	153 (38.2)	125 (37.8)	28 (40)		
	Red	142 (35.4)	113 (34.1)	29 (41.4)		
**Occupation, n (%)**						*<.001*
	Student	60 (15)	58 (17.5)	2 (2.9)		
	Employed	284 (70.8)	237 (71.6)	47 (67.1)		
	Retired	41 (10.2)	24 (7.3)	17 (24.3)		
	Unemployed	16 (4)	12 (3.6)	4 (5.7)		
**Risk associated with surgery, n (%)**						*.02*
	Minor	292 (72.8)	250 (75.5)	42 (60)		
	Intermediate	92 (22.9)	69 (20.8)	23 (32.9)		
	Major	17 (4.2)	12 (3.6)	5 (7.1)		
**Type of hospitalization, n (%)**						*<.01*
	Ambulatory	304 (75.8)	261 (78.9)	43 (61.4)		
	Hospitalization	96 (23.9)	69 (20.8)	27 (38.6)		
	N/A^c^	1 (0.2)	1 (0.3)	0 (0)		
**Previous teleconsultation, n (%)**						.94
	Yes	70 (17.5)	58 (17.5)	12 (17.1)		
	No	331 (82.5)	273 (82.5)	58 (82.9)		
Distance from home to the hospital (km), mean (SD)		66 (96)	70 (101)	50 (70)	.24	

^a^*P* values indicate comparisons between the teleconsultation and in-person consultation groups.

^b^ASA: American Society of Anesthesiologists.

^c^N/A: not available

### User Satisfaction

Patient satisfaction surveys revealed a median score of 9 (IQR 8-10) and 9 (IQR 8-9) for the teleconsultation and in-person consultation groups, respectively (*P*=.64 for group comparison). Only 13.6% (45/331) of patients in the teleconsultation group and 18.6% (13/70) in the in-person consultation group expressed a preference for having their next PAC in person.

### Perioperative Complications

In total, 4.1% (16/391) of the patients experienced complications within the first 6 postoperative months, without difference between groups (*P*=.24): 3.3% (11/331) of the patients had a PAC via teleconsultation, and 7.1% (5/70) of the patients were seen in in-person consultations.

The complications were predominantly infectious (10/391, 2.6%) and hemorrhagic (5/391, 1.3%). No patients required intensive care or died.

### Transportation Evaluation

The most frequently used mode of transportation for in-person consultations was a car (292/401, 72.8%). The mean distance between the patients’ residences and the university hospital was 66 (SD 96) km, with no statistically significant difference (*P*=.46) between groups. Nearly half (191/401, 47.6%) of the patients lived within 25 km, whereas 21.9% (88/401) resided more than 100 km from the hospital (Table 2).

**Table 2:** Distance and mode of transportation by type of preanesthetic consultation (hypothetical for teleconsultations; N=401).a

		Total, n (%)	Teleconsultations (n=331), n (%)	In-person consultations (n=70), n (%)	*P* value	
**Distance (km)**						
	<25	191 (47.6)	159 (48)	32 (45.7)	.32	
	25-50	58 (14.5)	41 (12.4)	17 (24.3)	.37	
	51-100	64 (16)	52 (15.7)	12 (17.1)	.56	
	101-200	65 (16.2)	57 (17.2)	8 (11.4)	.62	
	201-300	13 (3.2)	13 (3.9)	0 (0)		
	≥300	10 (2.5)	9 (2.7)	1 (1.4)		
**Mode of transportation**						*<.001*
	Car	292 (72.8)	252 (76.1)	40 (57.1)		
	Ambulance	33 (8.2)	19 (5.7)	14 (20)		
	Public transport	63 (15.7)	48 (14.5)	15 (21.4)		
	Bicycle or scooter	11 (2.7)	11 (3.3)	0 (0)		
	Plane	2 (0.5)	1 (0.3)	1 (1.4)		

^a^Mode of transportation used to attend the in-person consultations (car, medical transport, public transportation, bicycle, or scooter), distance between the home and the hospital, presence of a companion, and whether the patient or companion needed to take a full or half day off from work. For this purpose, patients who had undergone teleconsultations were asked to answer these questions hypothetically as if they had attended an in-person consultation (“If you had attended in person...”).

When comparing the type of transportation used by in-person consultation patients with the hypothetical mode of transport that teleconsultation patients would have used for in-person consultations, a statistically significant difference was observed (*P*<.001). Compared to in-person consultation patients, teleconsultation patients were more likely to use their personal car and less likely to use an ambulance (Table 2).

There was no significant difference in distance traveled based on the type of transport (not shown). Overall, teleconsultations reduced the total distance traveled by 44,266 km.

### Expenditure Evaluation

#### For Patients

#### Financial Savings

One patient traveling by air was excluded from the financial analyses due to the variability of airfare costs. The cost of a round trip was estimated per patient at €85 (SD €100) by car (252/331, 75.5%), €82 (SD €61) by ambulance (19/331, 5.7%), €3.3 (SD €0.70) by public transport (48/331, 14.5%), and €0 by bicycle or scooter (11/331, 3.3%). By avoiding travel, teleconsultations saved patients a total of €23,240 in travel costs (excluding parking). At our university hospital, patients benefit from a fixed parking fee of €1.80 per visit. Including parking, teleconsultations resulted in savings of €87 (SD €102) per patient using a car, totaling €23,694 in overall transportation savings.

More than a third of teleconsultation patients would have had to take time off work to attend an in-person consultation. In addition, 42% (139/331) would have been accompanied by a relative, who, in nearly half (165/331,%) of the cases, would also have needed to take time off work. There was no difference between the groups regarding the presence of an accompanying person or the need to take time off work for either the patient or their companion. There was no statistically significant association between the presence of an accompanying person and patient age (odds ratio 0.989, 95% CI 0.975-1.00; *P*=.15).

The theoretical income loss was estimated at €122 per workday. Overall, teleconsultations resulted in total savings of €16,408 in income loss for 330 of the patients, including €6627 saved by the accompanying persons. For students, retirees, or unemployed patients seen in teleconsultations (94/331, 28.4%), the financial loss was estimated at €5 per hour, with an average saving of €9.60 (SD €8.40) per patient, totaling €905.

The overall savings for the teleconsultation group combining theoretical travel costs and income loss amounted to €40,102 for 330 patients (Table 3), corresponding to an average of €122 (SD €125) per patient.

**Table 3:** Total savings for a patient seen in teleconsultation according to the type of transport.a A conversion rate of 1 US $=€1.17 applies.

Mode of transportation	Total, n	Cumulative expenditure (€), mean (SD)	Cumulative expenditure total=€40,102
Car	252	142 (132)	35,815
Ambulance	19	117 (74)	2230
Public transport	48	39 (41)	1926
Bicycle or scooter	11	15 (26)	131

^a^Transportation costs by car were estimated based on the standard mileage allowance for vehicle use (order of February 15, 2021; Service-Public.fr) using the formula distance × 0.541 (average for a vehicle with horsepower of 60 to 150). This rate includes vehicle power, fuel consumption, depreciation, maintenance costs, tire wear, and insurance premiums. Transportation costs for medical vehicles were calculated based on the applicable rates for the Occitania region [15]. These include a base fee (averaged at €12.70), a patient pickup fee (€15.58), and a per-kilometer rate (€1.02). The patient’s copay was €4.00 for a round trip. Short-distance fees (under 18 km) and additional fees for ambulance transport were not considered to avoid overestimating results. Health insurance was assumed to reimburse 65%.

#### Time Savings

The average transport time saved by teleconsultation patients was 123 (SD 100) minutes. In the subset of patients who would have traveled by car, over half (152/252, 60.3%) would have spent at least 2 hours traveling. The average round trip would have taken 125 (SD 100) minutes. In contrast, the mean transport time for in-person consultation patients was 85 (SD 75) minutes (all transport modes combined).

For in-person consultations, the patients’ journey included administrative registration; waiting time; the consultation with the anesthesiologist; and a potential discussion with a nurse for additional tests, electrocardiograms, or supplementary planning. The total time dedicated to the PCA (ie, transportation+administrative processes+PCA) for both the in-person consultation and teleconsultation groups was 130 (SD 16) and 22 (SD 9) minutes, respectively (*P*<.001). The average transportation time was 85 (SD 19) minutes in the in-person consultation group. The average time spent at the hospital for in-person consultation patients was 45 (SD 13) minutes. The average time spent for the PCA was 17 (SD 3) minutes for teleconsultations and 20 (SD 3) minutes for in-person consultations (*P*<.001 for group comparison), whereas the administrative time was longer for in-person consultations (mean 25, SD 4 minutes) compared to teleconsultations (mean 5, SD 3 minutes; *P*<.001).

#### For the National Health Insurance

Medical transportation services (ambulance) would have been used by 5.7% (19/331) of the patients in the teleconsultation group. The mean distance traveled was 89 (SD 83) km. In the teleconsultation group, the transportation cost per patient for a round trip was €226 (SD €174), with a reimbursement rate of 65% by the national health insurance, amounting to €144 (SD €113) per patient. Teleconsultations allowed the national health insurance to save €2737 for this 5.7% (19/331) of the patients. The total savings (patients+national health insurance) achieved in the teleconsultation group amounted to €42,840, corresponding to overall savings of €130 (SD €134) per patient.

#### For the Hospital

#### Specialized Software

The funding of TéléO by the Occitania Regional Health Agency did not result in any additional costs related to the use of this specialized software by the hospital.

#### Carbon Footprint Assessment

### Carbon Footprint Related to Transportation

By reducing travel, teleconsultations enabled a reduction in GHG emissions by 9688 kg CO_2_eq (Table 4).

**Table 4:** Carbon impact by mode of transport in the teleconsultation group.^a^

Mode of transport	Distance round trip saved (km)	CO_2_eq^b^ reduction (kg)
Car	39,792	8675
Ambulance	3386	738
Public transport	1088	2.6
Plane	1928	272
Bicycle	20	0
Total	46,214	9688

^a^For passenger vehicles, a medium-sized car with various types of engines (excluding electric and hybrid) was considered, with an emission factor of 0.218 kg of carbon dioxide equivalent per kilometer. Medical transport was considered to have the same emission factor. Public transport emissions were calculated assuming metro or tramway use, with an average factor of 0.0024 kg of carbon dioxide equivalent per kilometer. Bus use was not considered to avoid overestimating the results. Flights were considered short haul, with an emission factor of 0.414 kg of carbon dioxide equivalent per kilometer.

^b^CO_2_eq: carbon dioxide equivalent.

### Carbon Footprint Related to Teleconsultations

During an in-person PCA, the anesthesiologist systematically uses computer equipment to access the patient’s electronic medical records (ORBIS; Dedalus). The GHG emissions from medical computer equipment were calculated to be 0.004 kg CO_2_eq per consultation, corresponding to 0.096 kg CO_2_eq per consultation day. For teleconsultations, the digital connection between the patient and physician generated GHG emissions, detailed in Table 5. The total carbon footprint associated with digital connections was 2.30 kg CO_2_eq for the 331 teleconsultations conducted (Table 5). Owing to the remote consultation model for the 82.5% (331/401) of patients analyzed who underwent teleconsultations, the anesthesia department was able to reduce its total carbon emissions by 9686 tons of CO_2_eq, equating to a reduction of 29.3 kg CO_2_eq per patient.

**Table 5:** Energy consumption and carbon impact by mode of connection in the teleconsultation group.^a^

Mode of connection	Energy consumption (Wh)		CO_2_eq^b^ impact (kg)
	Patient	Anesthesiologist	

PC (n=244)	16,348	16,348	1.96
Smartphone (n=53)	3551	—^c^	0.21
Tablet (n=28)	1876	—	0.11
NA^d^ (n=6)	402	—	0.02
Total	22,177	16,348	2.30

^a^Energy consumption from IT equipment using fiber and digital subscriber lines was considered. A 5-minute connection to TéléO and a 20-minute teleconsultation session were assumed. Equipment used for teleconsultations included a fully equipped desktop computer (monitor, speakers, webcam, printer, and internet) consuming 200 W per hour of use, equivalent to 67 Wh for 20 minutes. Patient equipment energy consumption was only considered when using a computer (assumed to be a desktop) to avoid overestimation. Energy consumption from smartphones or tablets was not included as their use primarily affects manufacturing emissions rather than use.

^b^CO_2_eq: carbon dioxide equivalent.

^c^Not available.

^d^NA: not aplicable.

The main results of the economic and ecological impact evaluation of anesthesia teleconsultations are summarized in Table 6.

**Table 6:** Summary of results in the teleconsultation group. A conversion rate of 1 US $=€1.17 applies.

				Reduction per patient		Total reduction
**Economic impact**						
	**Transport savings (€**)**, mean (SD)**					
		For the patient	72 (94)		23,695 (27,827)	
		For the health insurance	144 (113)^a^		2737 (3214)	
	**Salary savings (€), mean (SD)**					
		All patients	30 (40)		9781(11,487)	
		Students, retirees, or unemployed patients	9.6 (8.4)		905 (1063)	
		Accompanying persons	20 (41)		6627 (7783)	
	Total savings (€), mean (SD)			130 (134)		42,840 (50,311)
	**Time savings (min)**					
		Transport, mean (SD)	123 (100)		34,082	
		Administrative registration	20^b^		6620	
		Consultation	3^c^		993	
		Total	146		48,326^d^	
	Round-trip distance savings (km), mean (SD)			140 (201)		46,214
**Ecological impact**						
	Transport-related CO_2_eq^e^ savings (kg)			29.3		9688

^a^Per patient supported by health insurance.

^b^Average administrative time for in-person consultations.

^c^Difference compared to the average consultation time for in-person consultations.

^d^Equivalent to 805 hours.

^e^CO_2_eq: carbon dioxide equivalent.

## Discussion

### Principal Findings

Through this study, we highlighted the medical, economic, and ecological benefits of conducting preanesthetic teleconsultations. Compared to in-person consultations, teleconsultations led to an average financial savings of €118 per patient, totaling over €42,000 in savings for 82.5% (331/401) of the patients. These economic benefits were primarily due to the reduction in travel-related expenses. Furthermore, approximately 10 tons of CO_2_eq emissions were avoided. Opting for home-based teleconsultations without paramedical facilitators could have limited the precision of clinical examinations compared to in-person consultations. However, most anesthetic risk assessments rely on scores obtained through patient interviews (Lee, metabolic equivalent of task, STOP-BANG, HEMSTOP, or APFEL scores). No correlation was found between the consultation modality and perioperative complications. When evaluating airways and intubation conditions, dental health, mouth opening, and the lip test were easily assessed via webcam, whereas the Mallampati score proved more challenging. Nevertheless, no significant differences in predicting difficult intubation were found between teleconsultations and in-person consultations for maxillofacial surgeries, although the positive predictive value for difficult intubation remained low in both cases [3]. Adjustments to airway management strategies following preanesthetic visits were consistent across both modalities.

In this study, patients expressed high satisfaction with teleconsultations, citing ease of use and intuitiveness. There was minimal “attachment” to in-person consultations. A study showed initial patient apprehension toward teleconsultations, with only half of the patients expressing willingness due to concerns about not meeting the physician, lack of equipment, or limited technical knowledge [18]. However, post-teleconsultation satisfaction is now well documented, with most patients reporting high satisfaction and a sense of security [3,19]. However, teleconsultations were not inferior to in-person consultations for preoperative patient evaluation and may be an interesting economic and ecological alternative [9]. During the COVID-19 pandemic, public health campaigns promoting confinement, the expansion of telecommunication technologies, and teleconsultation reimbursements likely influenced patient satisfaction. Technical issues (eg, poor network connectivity and lack of cameras or microphones) were the main limitations [20]. Improvements in technology and digital literacy are expected to enhance patient adherence, especially in fields such as orthopedic surgery where mobility challenges are significant [20].

Nearly 5000 patients are seen annually in PACs for planned orthopedic surgeries at Toulouse University Hospital. Given the hospital’s expertise, many patients must travel significant distances. Teleconsultations reduce travel time and distance, which is particularly beneficial in regions with limited mobility options.

For instance, a study found an average reduction of 447 km and 245 minutes per patient, saving over 8.6 million km and nearly 9 years of travel time over 17 years [21]. Another one noted an average saving of 102 km and 67 minutes per patient in the pediatric department in Kentucky, United States [22]. While these figures are not directly transferable to France, comparable results were observed in Portugal (47 km on average) [23]. In this study, teleconsultations reduced patient travel time 6-fold, aligning with the findings of previous studies [24].

Reduced travel also led to significant financial savings for patients. Similar findings have been reported in studies evaluating teleconsultation benefits. As an example, a minimum saving of US $32 per patient was noted in Arkansas, and average savings of US $150 per patient were observed in California [25]. This study found an average saving of €118 (SD €122) per patient [25,26]. The development of the child psychiatry teleconsultation program at the University of Kansas Medical Center has saved an average of US $86 per patient [27]. Over time, these savings accumulate; Dullet et al [21] estimated US $2.9 billion in savings over 17.5 years. In addition, one-third of patients in this study reported needing time off work for in-person consultations, aligning with the findings of Yen et al [28] that this applies to nearly half of patients. Patients living alone or geographically isolated would be the most likely to miss a day of work in the event of in-person consultations [25]. This loss of earnings is not negligible for the patient or their companion. Bynum et al [25] found a saving of US $75 to US $150 for 74% of the families of patients seen in teleconsultations, with reduced absenteeism at work. A Norwegian study suggested an average saving of €131 per patient by limiting absence from work [24], whereas our study estimated this saving at €122 per day. This study also highlights the potential for significant savings in health care reimbursements through teleconsultations. In this study, patients undergoing in-person consultations, often with a higher number of comorbidities, used sanitary vehicles more frequently, leading to higher transport costs [29]. Teleconsultations would help mitigate these expenses. Teleconsultations reduced the average consultation time without compromising patient satisfaction. This time saving could allow anesthesiologists to focus on complex cases or conduct additional consultations daily. Although not assessed in this study, previous research indicates no significant impact of teleconsultations on surgery cancellation rates even for major surgeries [30-32]. Patients living further from hospitals are more likely to cancel in-person appointments.

The health care sector contributes 3% to 8% of GHG emissions in high-income countries. To address these new environmental challenges, our health care services must undergo profound transformations. Among the strategies considered to reduce their ecological impact, hospitals are focusing on eHealth. Telemedicine is viewed as a promising technology, especially due to its potential to reduce transportation and air pollution. In 2021, patient transport was the second largest source of carbon emissions in health care following medication purchases. Digital health and teleconsultation are directly aligned with the ecological shift initiated by our specialty. Most studies on the environmental impact of teleconsultation only consider the reduction in transportation, which is the main source of carbon emissions. Dorrian et al [33], for example, highlighted a reduction of 123 kg CO_2_eq per patient for the 42 patients followed via teleconsultations for head and neck cancer. Masino et al [34] concluded that conducting 840 teleconsultations over 6 months in Ontario, Canada, led to a reduction in GHG emissions of 185 tons of CO_2_eq solely due to fewer travel requirements. The authors also remind us that carbon dioxide emissions are not the only GHG pollutants, and the emission of 360 kg of other airborne pollutants was also avoided. Over the 17 years of their program, Dullet et al [21] estimate a reduction in carbon emissions equivalent to the electricity consumption of 271 US households of 4 people. Similar results were found in studies from California, Scotland, and Wales [26,35,36]. In comparison, our study found a reduction of 9.7 tons of CO_2_eq owing to the implementation of teleconsultations, which represents an average saving of 29.3 kg CO_2_eq per patient. We also found that using public transport would have resulted in a CO_2_eq emission of 2.6 kg. Although our results strongly favor teleconsultations, the ecological impact of an in-person consultation could be significantly reduced through widespread use of public transport. Obviously, these conclusions are only applicable to urban populations with easy access to public transport networks. Nevertheless, these figures are significant in our study, where 47.6% (191/401) of the patients lived within 25 km of the hospital. However, the environmental trade-offs of the massive deployment of teleconsultations need to be evaluated in terms of a benefit-risk balance. Indeed, the IT infrastructure necessary for their implementation is a major source of carbon emissions. In 2021, IT represented 3% to 5% of the carbon footprint of an average university hospital. The emissions exclusively linked to the internal IT operations of public hospitals were estimated at over 190,000 tons of CO_2_eq annually, which is equivalent to 1 million 750 km round-trip flights for 1 person. The energy consumption of the IT sector is linked to 2 main areas: manufacturing (45%)—where the scope for action is limited except for rational purchasing—and use (55%). Use includes the operation of equipment (20%), data transmission via the internet (16%)—which is becoming more efficient and less energy consuming—and data storage in data centers (19%). Currently, only a minority of circulating health data are digitized. Their storage, which is expected to increase in both number and volume, and the associated carbon impact must prompt us to consider controlled IT consumption and digital sobriety. However, the carbon emissions linked to digital technologies for a 15-minute teleconsultation were minimal compared to the emissions avoided from travel. In fact, our study found an emission of 2.3 kg CO_2_eq for the 331 teleconsultations, representing a reduction of over 99% in carbon emissions compared to a traditional in-person consultation. Similar data were found during the COVID-19 pandemic [34,37]. These results are particularly noteworthy as the carbon impact of the IT sector remains relatively low in our country due to its largely decarbonized electricity production, with 92% of energy coming from nuclear, hydroelectric, solar, or wind sources.

Our study has several limitations. First, it was a single-center study. Even if our data are numerous, they can only apply to similar hospitals in similar areas. However, our results are consistent with the current literature on this topic. Second, due to lack of data, some simplifications and exclusions were made (such as type of transportation and underestimation of medical transport). In addition, for the estimation of ecological impact, only GHGs were considered following a simplified calculation approach rather than implementing a comprehensive environmental impact assessment method and life cycle assessment, among others. However, even underestimating some of the data, the results are in favor of teleconsultations. Finally, we did not consider electric or hybrid vehicles. Indeed, including electric vehicles would have required a more detailed energy system analysis, which is beyond the scope of this study.

### Conclusions

This retrospective study of 331 patients who underwent teleconsultations demonstrated a savings of over 46,000 km in travel distance, along with a reduction of nearly 10 tons of CO_2_eq emissions. In addition, significant savings in time spent on transportation and nonmedical hospital time were observed due to the use of teleconsultations. We were able to demonstrate a considerable direct financial benefit for the patient, as well as for the national health care system, through the reimbursement of medical transport costs. Moreover, our work highlighted the lack of attachment to in-person consultations by patients, as well as a high level of patient satisfaction with teleconsultations without any increase in postoperative complications. The medical-economic and ecological benefits of teleconsultations encourage us to refine and broaden the eligibility criteria for patients to use this mode of PAC.

## Abbreviations

CO2eq: carbon dioxide equivalent
GHG: greenhouse gas
PAC: preanesthetic consultation

## Data Availability

The datasets generated or analyzed during this study are available from the corresponding author on reasonable request.
